# Clinical Impact of Viral Load on the Development of Hepatocellular Carcinoma and Liver-Related Mortality in Patients with Hepatitis C Virus Infection

**DOI:** 10.1155/2016/7476231

**Published:** 2016-08-30

**Authors:** Ran Noh, Doo Hyuck Lee, Byoung Woon Kwon, Yong Hyun Kim, Suk Bae Kim, Il Han Song

**Affiliations:** Division of Hepatology, Department of Internal Medicine, Dankook University College of Medicine, Dankook University Hospital, Cheonan 330-715, Republic of Korea

## Abstract

*Aim*. This study aimed to assess clinical impact of hepatitis C viral load on the development of hepatocellular carcinoma (HCC) and liver-related mortality in HCV-infected patients.* Methods*. A total of 111 subjects with chronic HCV infection who were available for serum quantitation of HCV RNA were recruited in this retrospective cohort. Cox-proportional hazards models were used to calculate hazard ratio (HR) of developing HCC and liver-related mortality according to serum HCV RNA titers.* Results*. HCC was developed in 14 patients during follow-up period. The cumulative risk of HCC development was higher in subjects with high HCV RNA titer (log HCV RNA IU/mL > 6) than subjects with low titer (log HCV RNA IU/mL ≦ 6) (HR = 4.63, *P* = 0.032), giving an incidence rate of 474.1 and 111.5 per 10,000 person-years, respectively. Old age (HR = 9.71, *P* = 0.014), accompanying cirrhosis (HR = 19.34, *P* = 0.004), and low platelet count (HR = 13.97, *P* = 0.009) were other independent risk factors for the development of HCC. Liver-related death occurred in 7 patients. Accompanying cirrhosis (HR = 6.13, *P* = 0.012) and low albumin level (HR = 9.17, *P* = 0.002), but not HCV RNA titer, were significant risk factors related to liver-related mortality.* Conclusion*. Serum HCV RNA titer may be considered an independent risk factor for the development of HCC but not liver-related mortality.

## 1. Introduction

Hepatitis C virus (HCV) infection is one of the leading causes of chronic hepatitis worldwide and is also a major risk factor for the disease progression to advanced entities such as liver cirrhosis, hepatic decompensation, and hepatocellular carcinoma (HCC) [1, 2]. According to WHO estimates, approximately 3% of the world population or 170 million people may be infected with HCV [3, 4]. After the introduction of the hepatitis B virus (HBV) vaccination program, HCV-related disease burdens have been progressively emerging in HBV-endemic areas [5, 6]. In Korea, chronic liver diseases including chronic hepatitis, liver cirrhosis, and HCC accounted for approximately 7.1% of the total deaths in 2010 with a mortality rate of 46.3/100,000 people. It is a leading cause of death for men in their forties and fifties, and mortality due to HCC amounts to 15.6% of the total cancer deaths [7]. There are several factors affecting the clinical course of hepatitis virus-infected patients: host factors, virologic factors, and environmental factors. Among them, virologic factors have been investigated to a large extent in cases with hepatitis virus-related liver cirrhosis or HCC. In HBV-infected patients, the clinical implications of serum HBV DNA levels for liver disease progression are enough to be recognized. A stepwise increase of serum HBV DNA level has been determined to be associated with a corresponding linear increase in the cumulative incidence of HCC as well as the progression of HBV-related liver disease to liver cirrhosis or hepatic decompensation, regardless of serum transaminase activity, hepatitis B e antigen (HBeAg) serostatus, and the current presence of cirrhosis [8–10]. Also it is well known that inactive HBV carriers are still at risk for HCC and liver-related death [11]. Furthermore, spontaneous seroclearance of HBV DNA is known to be the most significant factor in reducing risk for future HCC [12]. Namely, serum HBV DNA level itself is a major independent risk factor affecting the development of HCC and liver-related mortality. However, the influence of HCV on the development of HCC and liver-related death in HCV-infected patients is uncertain. In particular, whether liver disease progression is HCV RNA titer-dependent remains to be determined. Therefore, we tried to assess the clinical impact of HCV viral load on the development of HCC and liver-related mortality in patients with chronic HCV infection.

## 2. Methods

### 2.1. Study Subjects

**Figure 1:**
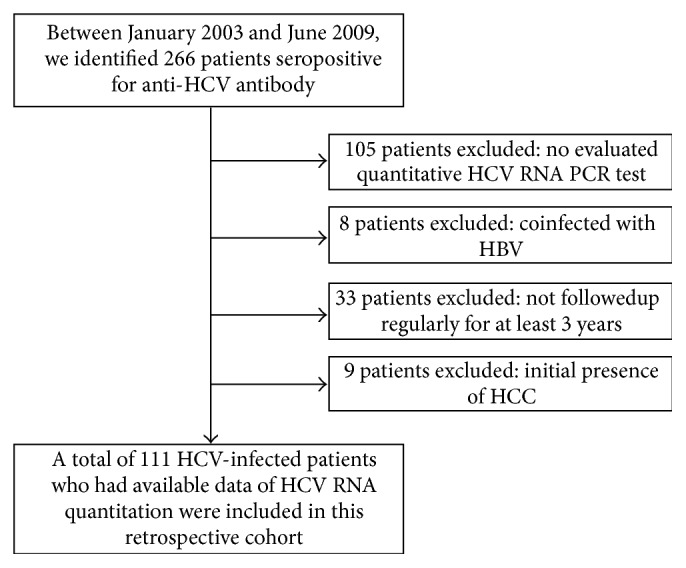
Schematic flow of the recruitment of study participants.

This study was retrospectively conducted by reviewing medical records. We identified 266 patients seropositive for anti-HCV antibody between January 2003 and June 2009 at Dankook University Hospital in Korea. Among them, 155 patients were excluded under the following exclusion criteria: (1) 105 patients whose quantitative tests of serum HCV RNA were not evaluated, (2) 8 patients who were coinfected with HBV, (3) 33 patients who were not followed up regularly for at least 3 years, and (4) 9 patients initially presenting with HCC. Ultimately, a total of 111 HCV-infected patients who had available data of serum HCV RNA quantitation were recruited in this retrospective cohort. The schematic flow for the enrollment of study participants is shown in Figure 1. Clinical characteristics and demographics of enrolled patients including age, gender, accompanying cirrhosis at the recruitment time, and follow-up duration from the enrollment were obtained from the medical records. Laboratory findings such as liver tests, serum HCV RNA titer, and HCV genotype were included in this study analysis. We also ascertained whether antiviral therapies were performed and whether sustained virologic responses were achieved during the follow-up period. The present study was conducted in accordance and compliance with the ethical principles of the 1975 Declaration of Helsinki.

### 2.2. HCV-Related Laboratory Examinations

Anti-HCV antibodies were detected using an immunoradiometric assay (Shin Jin Medics, Inc., Goyang, Korea) based on recombinant proteins from the HCV genome, which showed a sensitivity of 98.5% and a specificity of 99.7%. Serum HCV RNA titers were quantitatively determined by real-time polymerase chain reaction (PCR) using COBAS TaqMan HCV quantitative test, version 2.0 (Roche Molecular Systems, Inc., Branchburg, NJ, USA) with a linear range from 43 to 69,000,000 IU/mL. In the present study, we classified patients into two groups with high or low viral load based on the serum HCV RNA level at study entry. The cut-off log titer of HCV RNA levels was 6: (1) high viral load > 6 log titer of HCV RNA IU/mL and (2) low viral load ≦ 6 log titer of HCV RNA IU/mL. HCV genotyping was performed by reverse transcription PCR and sequencing analysis using ABI PRISM 3130 Genetic Analyzer (Life Technologies, Carlsbad, CA, USA).

### 2.3. Ascertainment of HCC and Liver-Related Death

Hepatocellular carcinoma was confirmed by histology. Cases not available for histologic evaluation were diagnosed by applying nonhistologic criteria, mainly depending on the radiologic imaging on the base of chronic liver diseases. The radiologic findings typical of HCC diagnosis are an enhancement of a liver nodule on the arterial phase with a washout on the portal or delayed venous phase. Nonhistologic criteria of the HCC diagnosis were adopted by two dynamic image modalities (e.g., abdominal computed tomography and magnetic resonance imaging) suitable to radiologic findings of HCC mentioned above, or by one imaging modality appropriate to HCC plus a serum *α*-fetoprotein level ≧ 400 ng/mL [13]. In high risk patients with liver cirrhosis, the dynamic imaging criteria updated in the 2010 American Association for the Study of Liver Diseases guideline were also applied to ascertain the development of HCC by reviewing medical records and imaging modalities [14]. Liver-related death was defined as the primary cause of death due to complications of liver cirrhosis, hepatic failure, or HCC.

### 2.4. Statistical Analysis

Cumulative incidence rates of HCC and liver-related death were calculated by the Kaplan-Meier method with a log-rank test to verify the significance. Person-years of follow-up for each subject were calculated as the time period from the date of clinical baseline survey at study enrollment to the date of HCC diagnosis, the date of death, or the date of last follow-up or at least June 30, 2012, whichever occurred first. Cox proportional-hazards regression was used for univariate analysis and multivariate analysis to determine the most significant and independent variables related to the development of HCC and liver-related mortality. Hazard ratios (HR) and corresponding 95% confidence intervals (95% CI) were calculated to estimate the degree of association between risk variables and the development of HCC or liver-related death. Statistical analyses were performed using SPSS Statistics version 18.0.* P *< 0.05 were considered as a significant value.

## 3. Result

### 3.1. Patient Characteristics

The clinical characteristics of 111 HCV-infected patients (54 males, 57 females) are listed in Table 1. The mean age at the study entry was 53 ± 13 years. Twenty-nine (26.1%) patients initially presented with liver cirrhosis. The number of patients with high viral load (log titer of HCV RNA IU/mL > 6) and low viral load (log titer of HCV RNA IU/mL ≦ 6), based on serum HCV RNA titer, were 52 (46.8%) and 59 (53.2%), respectively. Thirty-nine (52%) of 75 patients who were available for HCV genotyping had genotype 1, the most prevalent in Korea. During the follow-up period, 43 (38.7%) patients received antiviral therapy with a global protocol of peginterferon and ribavirin as the standard of care. Namely, HCV genotype 1 patients received a combination treatment of peginterferon-alpha and ribavirin for 48 weeks, and HCV genotype non-1 patients received the same regimen for 24 weeks. Among them, 31 (72%) obtained a sustained virologic response. After study entry, all patients were followed-up for 54 ± 16 months of mean duration.

### 3.2. Development of HCC

**Figure 2:**
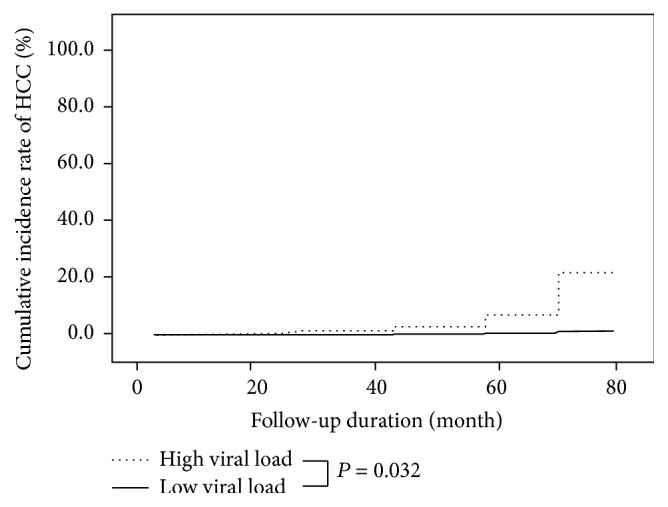
Cumulative incidence of hepatocellular carcinoma according to the viral load of serum HCV RNA. The cumulative incidence rate of HCC in patients with high viral load (log HCV RNA IU/mL > 6) was significantly higher than that in patients with low viral load (log HCV RNA IU/mL ≦ 6) (*P* = 0.032).

HCC was newly developed in 14 patients during the follow-up period. On univariate analysis, old age (≧50 years old), initial presence of cirrhosis, high viral load of serum HCV RNA (>6 log titer of HCV RNA IU/mL), low platelet count (<130,000/mm^3^), and low serum albumin level (<3.0 g/dL) were significant risk factors associated with the development of HCC. Clinical factors with *P* value less than 0.05 on the univariate analysis were incorporated into a multivariate analysis. The risk of HCC development was higher in subjects with high viral load of HCV RNA than subjects with low viral load (HR = 4.63, *P* = 0.032) (Table 2). The five-year cumulative incidence rate of HCC in patients with high viral load was 6.8%, being significantly different compared to 1.7% of patients with low viral load (Figure 2). Old age (HR = 9.71,* P* = 0.014), initial presence of cirrhosis (HR = 19.34, *P* = 0.004), and low platelet count (HR = 13.97, *P* = 0.009) were other independent risk factors for the cumulative incidence of HCC (Table 2).  Table 3 displayed the incidence rates of HCC per 10,000 person-years by significant risk factors analyzed above. The incidence rate of HCC per 10,000 person-years in patients with high viral load was higher than ones with low viral load (474.1 versus 111.5).

### 3.3. Development of Liver-Related Death

**Figure 3:**
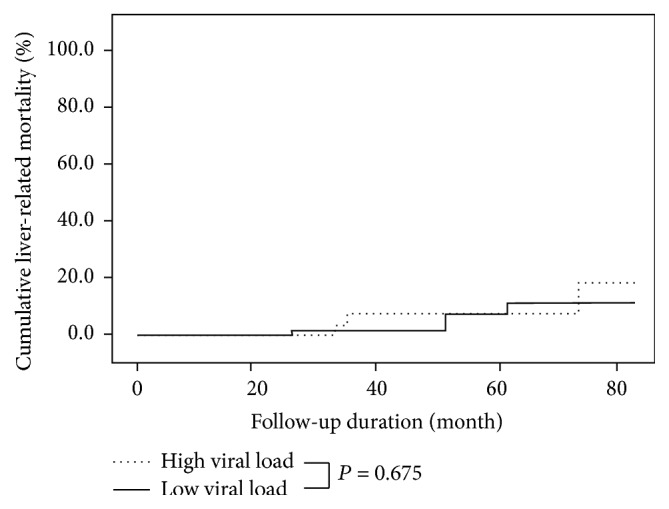
Cumulative liver-related mortality according to the viral load of serum HCV RNA. No significant difference of cumulative liver-related mortality was seen in patients with high or low viral load (*P* = 0.675).

A total of eight patients died during follow-up period. Among them, liver-related death occurred in 7 patients and the remaining patient died of a lymphoma-associated complication. The causes of liver-related death were 3 cases with liver failure, 3 cases with HCC, and 1 case with bleeding esophageal varices. Initial presence of cirrhosis, low platelet count (<130,000/mm^3^), and low serum albumin level (<3.0 g/dL) were significant risk factors associated with liver-related mortality on univariate analysis. However, the viral load of serum HCV RNA was not significant. Multivariate analysis with Cox-proportional hazards models finally indicated initial presence of cirrhosis (HR = 6.13, *P* = 0.012) and low serum albumin level (HR = 9.17, *P* = 0.002) as significant risk factors for liver-related death (Table 4). There was no difference of cumulative liver-related mortality according to the serum level of HCV RNA. Hepatitis C viral load itself did not affect liver-related death in this study (Figure 3).

## 4. Discussion

In this retrospective cohort study, we intended to assess the clinical impact of hepatitis C viral load on the development of HCC and liver-related mortality in HCV-infected patients. As a result, the present study revealed that the quantitative level of serum HCV RNA was an independent risk factor for the development of HCC, although HCV viral load itself did not affect liver-related death.

It has been speculated that higher HCV RNA titers might lead to a higher incidence of HCC. However, to our knowledge, the influence of hepatitis C viral load on the development of HCC has been controversial in previous studies. Furthermore, little was reported about the clinical implications of hepatitis C viral load with regard to liver-related death. The impact of viral load of hepatitis C on the incidence of HCC was investigated through a large-scaled population-based cohort in Japan [15]. The result demonstrated that HCV viremia was strongly associated with the occurrence of HCC without virus titer dependence. But because HCV core antigen, instead of HCV RNA, was measured to quantify HCV, the nucleic acid-based clinical impact could not be exactly assessed in that study. In a community-based long-term follow-up study from Taiwan, the risk of HCC stepwise increased significantly from the subjects seronegative for HCV RNA to subjects with low HCV RNA titer and to subjects with high HCV RNA titer, which indicated strong implications for the management of chronic HCV infection based on elevated serum HCV RNA level as an independent risk predictor of HCC [16]. However, several previous studies failed to find an association between serum HCV RNA levels and the risk of HCC [17–19]. Our results revealed that the cumulative risk of HCC development was higher in patients with high titer of HCV RNA than patients with low titer, giving an incidence rate of 474.1 and 111.5 per 10,000 person-years, respectively. As a result, we consider a serum HCV RNA titer as an independent risk factor for the development of HCC.

It is an important matter to determine the threshold value of viral load associated with an increased risk of long-term adverse outcomes in HCV-infected patients. Currently, clinical studies for cut-off serum level of hepatitis C viral load relevant to the development of HCV-related HCC are rare. In one study, the median detectable serum HCV RNA levels (3.5 × 10^5^ U/mL) at the study entry were based on classifying study participants into subjects with high viral load or low viral load [16]. The other study classified participants into two groups: high (≧10^5^ copies/mL) and low (<10^5^ copies/mL) viral load [20]. In our analysis, the mean log titer of serum HCV RNA level (IU/mL) in 111 HCV-infected patients was 5.7. Based on the mean log titer of HCV RNA, we classified study subjects into two groups: high viral load group (>6 log titer of HCV RNA) and low viral load group (≦6 log titer of HCV RNA), resulting in the obtainment of similar sample sizes and no difference of baseline characteristics between the two groups.

In addition to HCV RNA viral load, in the present study, old age, low platelet count, and the presence of cirrhosis were found to be significant and independent risk factors for the development of HCC. Although the patient age ≧ 50 at the study entry was associated with HCC development, the period from the diagnosis of HCV infection would be more important than the patient age at the study recruitment, as shown in case of HBV infection. However, it was difficult to know exactly the time period from HCV infection to study recruitment of subjects because of the innate limitation of this retrospective study. Several studies demonstrated that HCV genotype 1b was more closely related to the development of HCC than the genotype non-1b [15, 16, 21–24], but there have been disagreements against this aspect [25–27]. In the present study, genotype 1b was not analyzed to be an independent risk factor of HCC by the analysis of Cox-proportional hazard model, even if genotype 1b had a tendency to result in more frequent development of HCC than non-1b genotype (HR = 0.32, *P* = 0.185). This result should be further evaluated because HCV genotypes in our study were examined in a small proportion of study subjects who were in need of anti-HCV therapy. A meta-analysis of controlled trials has shown that antiviral therapy reduced the rate of HCC development in patients with type C liver cirrhosis [28]. Previous reports have shown that sustained virologic response (SVR) suppresses HCC occurrence in patients with HCV-related liver cirrhosis and chronic hepatitis [29–31]. Recently, other studies have reported that peginterferon-based antiviral therapy does not reduce the incidence of HCC among patients with advanced HCV chronic liver disease who did not achieve SVR even though a potential clinical benefit of long-term suppressive therapy was expected [32–34]. In the present study, clinical impact according to whether or not antiviral therapy was received and subsequently SVR was acquired did not reach statistical significance to indicating independent risk factors of HCC development. This finding should be confirmed in a large-scaled prospective study with subjects involved in anti-HCV therapy.

SVR is known to be associated with not only the inhibition of HCC development, but also the control of all-cause mortality and liver-related mortality in chronically HCV-infected patients [35, 36]; furthermore, low HCV viral load was reported to predict better long-term outcomes including 5-year survival rate in patients undergoing resection of HCC regardless of serologic eradication of HCV [37]. However, no available data regarding a causal relationship between the serum titer of HCV RNA and liver-related death have been reported. In this study, factors that affect liver-related death were serum albumin level and the initial presence of liver cirrhosis, but not HCV viral load, indicating that liver-related mortality in HCV-infected patients seems to be mainly related to hepatic reserve function. Actually, causes of death in mortality cases of this study resulted from hepatocellular insufficiency or portal hypertension such as liver failure, HCC, and bleeding esophageal varices. Recently, Cramp et al. [38] suggested that, with the current treatment practice, the number of patients developing HCV-related cirrhosis, hepatic decompensation, and HCC will increase substantially, with HCV-related liver death likely to double by 2030. It is time to challenge diagnostic and therapeutic strategies to optimize the reduction in the burden of HCV-related diseases.

There were several limitations that could not be averted in this study. First, this is a single-center, hospital-based cohort study with a small sample size and relatively short follow-up duration. So, multicenter, population-based long-term follow-up data are needed to strengthen and generalize the study results. Second, because the study was retrospective in nature, we could not escape unmeasured or unrecorded clinical data conducting the statistical analyses. Third, we could not consider suggestive baseline characteristics, such as body mass index, amount of alcohol consumption, or presence of diabetes mellitus, which are considered to be significant risk factors predicting the development of HCC in patients with chronic HCV infection. Fourth, the serum level of HCV RNA is a dynamic parameter in patients with chronic hepatitis C; however, we presented the measurement of viral load from a study entry point in time, which could not give the complete picture of the association of the severity of the liver disease based on the serologic fluctuation of viral load. Although the influence of these limitations on our results would not be substantial, these considerations indicate the need for care in interpretation of these results.

## 5. Conclusion

This retrospective cohort study demonstrated that the serum HCV RNA titer may be considered to be an independent risk factor affecting the development of HCC but not liver-related mortality in HCV-infected patients. Although any interpretation of these results requires careful consideration, the establishment of risk factors for HCC, such as hepatitis C viral load, may be useful in determining a follow-up strategy in HCV-infected patients. Furthermore, large-scaled long-term follow-up studies to assess whether the present results can be generalized or are representative of other populations are needed.

## Figures and Tables

**Table 1:** Baseline characteristics of enrolled subjects.

Characteristics	Values
Age, year	53 ± 13

Male gender, *n* (%)	54 (48.6)

Initial presence of cirrhosis, *n* (%)	29 (26.1)

Child-Pugh classification, *n* (%)	
A	78 (70.3)
B	26 (23.4)
C	7 (6.3)

Alanine aminotransferase, IU/L	80 ± 84

Bilirubin, mg/dL	1.2 ± 1.8

Prothrombin time, INR	1.2 ± 0.3

Platelet, ×10^3^/mm^3^	164 ± 67

Albumin, g/dL	4.1 ± 0.6

Creatinine, mg/dL	0.9 ± 1.2

HCV RNA (log IU/mL), *n* (%)	
Low (≦6log⁡)	59 (53.2)
High (>6log⁡)	52 (46.8)

HCV genotype, *n* (%)	
1b	39/75 (52.0)
Non-1b	36/75 (48.0)
Not checked	36/111 (32.4)

Antiviral therapy, *n* (%)	
No	68/111 (61.3)
Yes	43/111 (38.7)

Sustained virologic response, *n* (%)	
No	12/43 (28.0)
Yes	31/43 (72.0)

Follow-up duration, months	54 ± 16

Data are the number (percentage) or mean ± standard deviation.

HCV: hepatitis C virus.

**Table 2:** Analysis of variables associated with the development of hepatocellular carcinoma.

Variables	Univariate analysis hazard ratio (95% CI)	*P* value	Multivariate analysis hazard ratio (95% CI)	*P* value
Age, year (<50 versus ≧50)	11.07 (2.01–62.42)	0.006	9.71 (1.03–39.19)	0.014

Sex (female versus male)	0.94 (0.89–1.29)	0.914		

Initial presence of cirrhosis (no versus yes)	28.24 (3.87–205.43)	<0.001	19.34 (2.26–165.07)	0.004

Serum HCV RNA titer, log IU/mL (≦6 versus >6)	5.01 (1.46–26.47)	0.018	4.63 (1.14–18.88)	0.032

HCV genotype (non-1b versus 1b)	0.32 (0.26–1.07)	0.185		

Platelet, ×10^3^/mm^3^ (≧130 versus <130)	19.97 (3.32–86.12)	<0.001	13.97 (1.96–68.99)	0.009

Alanine aminotransferase, IU/mL (<40 versus ≧40)	1.00 (0.97–1.02)	0.833		

Prothrombin time, INR (<1.2 versus ≧1.2)	2.52 (1.86–2.99)	0.304		

Creatinine, mg/dL (<1.2 versus ≧1.2)	0.95 (0.91–1.01)	0.827		

Bilirubin, mg/dL (<1.5 versus ≧1.5)	0.97 (0.92–1.00)	0.864		

Albumin, g/dL (≧3.0 versus <3.0)	3.28 (2.46–6.63)	0.030	1.154 (1.072–5.198)	0.165

History of antiviral therapy (yes versus no)	0.23 (0.35–1.01)	0.061		

Sustained virologic response (yes versus no)	0.17 (0.34–1.00)	0.097		

Significant variables in the univariate analysis were incorporated into a multivariate analysis.

HCV: hepatitis C virus.

**Table 3:** Incidence rates of hepatocellular carcinoma per 10,000 person-years by significant variables.

Variables	Patients (*n* = 111)	Number of HCC	Incidence rate of HCC per 10,000 person-years
Age, years			
<50	45	1	47.6
≧50	66	13	446.7

Initial presence of cirrhosis			
No	82	2	52.6
Yes	29	12	1000

Platelet, ×10^3^/mm^3^			
≧130	76	2	57.5
<130	35	12	784.3

Serum HCV RNA titer			
Low (≦6log⁡)	59	3	111.5
High (>6log⁡)	52	11	474.1

HCV: hepatitis C virus.

**Table 4:** Analysis of variables associated with liver-related mortality.

Variables	Univariate analysis hazard ratio (95% CI)	*P* value	Multivariate analysis hazard ratio (95% CI)	*P* value
Age, year (<50 versus ≧50)	1.02 (1.01–1.49)	0.605		

Sex (female versus male)	6.24 (2.94–9.12)	0.095		

Initial presence of cirrhosis (no versus yes)	8.33 (2.98–54.23)	0.015	6.13 (1.60–31.78)	0.012

Serum HCV RNA titer, log IU/mL (≦6 versus >6)	3.03 (1.79–5.39)	0.197		

HCV genotype (non-1b versus 1b)	0.01 (0.00–1.05)	0.998		

Platelet, ×10^3^/mm^3^ (≧130 versus <130)	2.99 (1.57–5.21)	0.048	1.23 (1.01–3.13)	0.191

Alanine aminotransferase, IU/mL (<40 versus ≧40)	0.99 (0.96–1.02)	0.357		

Prothrombin time, INR (<1.2 versus ≧1.2)	6.26 (3.63–11.01)	0.075		

Creatinine, mg/dL (<1.2 versus ≧1.2)	0.85 (0.71–1.01)	0.831		

Bilirubin, mg/dL (<1.5 versus ≧1.5)	1.12 (0.87–1.97)	0.378		

Albumin, g/dL (≧3.0 versus <3.0)	4.19 (1.89–68.41)	0.003	9.17 (1.02–48.51)	0.002

History of antiviral therapy (yes versus no)	0.25 (0.11–1.00)	0.202		

Sustained virologic response (yes versus no)	0.01 (0.00–1.04)	0.998		

Significant variables in the univariate analysis were incorporated into a multivariate analysis.

HCV: hepatitis C virus.

## Abbreviations

HCV: Hepatitis C virus
HCC: Hepatocellular carcinoma
HR: Hazard ratio
HBV: Hepatitis B virus
HBeAg: Hepatitis B e antigen
PCR: Polymerase chain reaction
CI: Confidence intervals
SVR: Sustained virologic response.
